# Predicting resilience of ecosystem functioning from co‐varying species' responses to environmental change

**DOI:** 10.1002/ece3.5679

**Published:** 2019-09-27

**Authors:** Matthew P. Greenwell, Tom Brereton, John C. Day, David B. Roy, Tom H. Oliver

**Affiliations:** ^1^ School of Biological Sciences University of Reading Reading UK; ^2^ Butterfly Conservation Wareham UK; ^3^ NERC Centre for Ecology & Hydrology Wallingford UK

**Keywords:** Ecosystem functioning, ecosystem resilience, effect traits, environmental change, environmental risk, population dynamics, response guilds, response traits

## Abstract

Understanding how environmental change affects ecosystem function delivery is of primary importance for fundamental and applied ecology. Current approaches focus on single environmental driver effects on communities, mediated by individual response traits. Data limitations present constraints in scaling up this approach to predict the impacts of multivariate environmental change on ecosystem functioning.

We present a more holistic approach to determine ecosystem function resilience, using long‐term monitoring data to analyze the aggregate impact of multiple historic environmental drivers on species' population dynamics. By assessing covariation in population dynamics between pairs of species, we identify which species respond most synchronously to environmental change and allocate species into “response guilds.” We then use “production functions” combining trait data to estimate the relative roles of species to ecosystem functions. We quantify the correlation between response guilds and production functions, assessing the resilience of ecosystem functioning to environmental change, with asynchronous dynamics of species in the same functional guild expected to lead to more stable ecosystem functioning.

Testing this method using data for butterflies collected over four decades in the United Kingdom, we find three ecosystem functions (resource provisioning, wildflower pollination, and aesthetic cultural value) appear relatively robust, with functionally important species dispersed across response guilds, suggesting more stable ecosystem functioning. Additionally, by relating genetic distances to response guilds we assess the heritability of responses to environmental change. Our results suggest it may be feasible to infer population responses of butterflies to environmental change based on phylogeny—a useful insight for conservation management of rare species with limited population monitoring data.

Our approach holds promise for overcoming the impasse in predicting the responses of ecosystem functions to environmental change. Quantifying co‐varying species' responses to multivariate environmental change should enable us to significantly advance our predictions of ecosystem function resilience and enable proactive ecosystem management.

## Graphical abstract

Current approaches to understand how environmental change affects ecosystem function delivery have reached an impasse due to data limitations and scaling constraints. Here, we present a more holistic approach using long‐term monitoring data to analyze the aggregate impact of multiple historic environmental drivers on species' population dynamics and what effects these have on ecosystem functioning. Quantifying co‐varying species' responses to multivariate environmental change should enable us to significantly advance our predictions of ecosystem function resilience and to enable proactive ecosystem management.

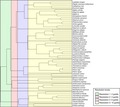

## 1 INTRODUCTION

Ecological systems are essential to human society for many reasons, including the provision of ecosystem functions and services (Díaz et al., 2013). These services include regulation of climate, prevention of flooding, provision of resources and cultural well‐being (Costanza et al., 1997). A rapidly rising global population is leading to a growing demand for ecosystem services (Biggs et al., 2012); however, consequent anthropogenic drivers degrading ecosystems mean that their ability to deliver these services is increasingly at risk (Millennium Ecosystem Assessment, 2005; UK National Ecosystem Assessment, 2011). A key factor in the maintenance of ecosystem functions and services is biodiversity (Cardinale et al., 2012; Harrison et al., 2014; Hector & Bagchi, 2007; Isbell et al., 2011; Lefcheck et al., 2015). Human activities, including habitat fragmentation, pollution, and climate change, have led to declines in both species richness and abundance, as well as increased extinction risk (Newbold et al., 2015; Pimm et al., 2014; Tittensor et al., 2014).

Understanding how ecosystem services will respond to changes in species assemblages is regarded as an urgent priority for informing ecosystem management (De Palma, Dennis, Brereton, Leather, & Oliver, 2017; Díaz et al., 2013; Oliver et al., 2015). Indeed, the ability to predict ecological functions from species' traits has been hailed as the “Holy Grail” of functional ecology (Funk et al., 2017; Lavorel & Garnier, 2002; Suding & Goldstein, 2008). Yet, after decades of research, there is still limited ability to make predictions of multiple environmental drivers on ecosystem functioning for multiple species in real‐world situations. Previous attempts to predict the impact of environmental changes on ecosystem functions and services have focused on a “reductionist” approach, attempting to determine how ecological traits (“response traits”) mediate community responses to environmental change, and how altered community composition then leads to changes in ecosystem function delivery (mediated by species' “effect” traits; Díaz et al., 2013).

Since its introduction into ecological literature by Holling (1973), the use of the term resilience has encompassed a number of different definitions, leading to confusion and no clear consensus within the literature (Walker, Holling, Carpenter, & Kinzig, 2004). A key reason for this is that resilience can be split into ecological resilience, that is, the magnitude of disturbance that a system can experience before shifting into a different state, including the ability of a system to maintain its functioning, structure, and identity (Berkes, Colding, & Folke, 2003; Chappin, Kofinas, & Folke 2009; Elmqvist et al., 2007; Folke et al., 2004; Gunderson & Allen, 2010; Suding et al., 2008); aspects that are sometimes termed “resistance” (Donohue et al., 2013); and engineering resilience, that is, the time taken for a system to return to equilibrium after a perturbation (Holling, 1996; Pimm, 1984). While engineering resilience draws from a more classical use of the term outside of ecology, stemming from the etymology of the word (Gunderson & Allen, 2010), it should not be considered as the definitive term for resilience in ecology (Walker et al., 2004). It should also be noted that resilience, along with constancy, and persistence are factors that contribute to the overall stability of an ecosystem (Grimm & Wissel, 1997), which also encompasses a number of other factors including robustness and variability (Donohue et al., 2013). In this study, we focus specifically on the ability of an ecosystem function to be maintained in the face of environmental perturbations, therefore integrating aspects of resistance and adaptive capacity from Holling's (1973) definition of ecological resilience, and recovery from Pimm's (1984) engineering resilience definition. Sometimes, the same underlying mechanisms can be responsible for both resistance and recovery, and rapid recovery can appear as resistance depending on the time window of measurement (Oliver et al., 2015). Therefore, using resilience as an umbrella term for resistance and recovery makes good sense and is increasingly widely used by others (Beller et al., 2019; Kohler et al., 2017). Specifically, the term resilience hereon refers to “the degree to which an ecosystem function can resist or recover rapidly from environmental perturbations, thereby maintaining function above a socially acceptable level” (Oliver et al., 2015).

The resilience of any particular ecosystem function to a certain environmental driver is related to the correlation between response and effects traits (Díaz et al., 2013; Oliver et al., 2015; Suding et al., 2008). For example, if all species which are important pollinators of a certain crop are highly susceptible to warmer winters (i.e., positive correlation between response and effects traits), then crop pollination would have a low resilience to that aspect of environmental change. In contrast, a lack of correlation would lead to the maximum resilience of the ecosystem function (Díaz et al., 2013; Larsen, Williams, & Kremen, 2005).

There are, however, a number of significant limitations with this approach that constrain its applicability. Firstly, the number of species for which accurate trait data are available is severely limited, typically restricted to plant species (Kattge et al., 2011). Where trait data are available for other taxa, they tend to be “soft traits” such as body size, with tenuous or unknown correlations to environmental change and/or ecosystem functioning. There can also be significant disagreements regarding trait measurements between different datasets for the same species (Middleton‐Welling, Wade, Dennis, Dapporto, & Shreeve, 2018). Importantly, even where accurate trait data are available, trait‐based analyses cannot always be reliably transferred to different regions (Powney, Preston, Purvis, Van Landuyt, & Roy, 2014), and in many cases, the goodness of fit of the relationships between putative response traits and environmental change or between putative effect traits and ecosystem function is too low to be used predictively (Lavorel & Garnier, 2002; Luck, Lavorel, McIntyre, & Lumb, 2012).

In some cases, the same trait can be used as both the response and effect trait. For example, body size can be used as a response trait when investigating the effects of agricultural intensification on pollinators and can also be used as an effect trait to predict pollination efficiency (Larsen et al., 2005). Here, the ability to predict the effects of agricultural intensification on pollinators depends on two relationships: a regression of agricultural intensification on body size, and a regression of body size on pollination. Unfortunately, the goodness of fit for such relationships is often low (Lavorel & Garnier, 2002; Luck et al., 2012). Furthermore, in the majority of cases, a different effect trait must be used from the response trait meaning an additional relationship between the two traits must be calculated, adding further uncertainty and reducing the predictive power of the models.

The substantial sources of uncertainty severely constrain our ability to predict the delivery of ecosystem functions under any particular aspect of environmental change. It may explain why the few successful demonstrations have been limited to studying plant communities (Lavorel et al., 2011), with most focusing on single ecosystem functions (primary regulating services), and only 11% of studies considering more than two ecosystem functions (Hevia et al., 2017). Furthermore, only 4% of trait‐based approaches consider the simultaneous effects of multiple environmental drivers (Hevia et al., 2017), even though we know that drivers such as climate and land use change strongly interact in their impacts on biodiversity (Brook, Sodhi, & Bradshaw, 2008; Oliver & Morecroft, 2014). We expect the environment to change across multiple variables (e.g., multiple different aspects of climate and land use change); therefore, additively combining predictions of the effects of single drivers in order to understand the effects of multiple drivers on general resilience of ecosystem functioning makes the overall uncertainty in these reductionist predictive frameworks untenable.

These problems may explain the apparent impasse in functional ecology whereby attempts to develop a predictive framework using a reductionist “Holy Grail” approach have been ongoing since the late 1990s (Díaz & Cabido, 1997; Lavorel, McIntyre, Landsberg, & Forbes, 1997), with revisits in the early 2000s (Lavorel & Garnier, 2002), and again more recently (Funk et al., 2017). After decades of methodological development with only limited application (Gross et al., 2008; Suding & Goldstein, 2008), new methods are urgently needed to predict the resilience of ecosystem functioning under environmental change.

Here, we propose a more holistic approach, utilizing long‐term population monitoring data that reflect the aggregate effects of multivariate environmental change on species' population dynamics. Using this method, groups of species with similar responses to multiple historic environmental drivers, identified through more synchronous population dynamics, can be allocated into “response guilds.” The distribution of effects traits across these response guilds can then inform on the resilience of ecosystem functioning.

Changes in population dynamics are due to the interactions between organisms and the combined biotic and abiotic effects of their environments (Wallner, 1987). Covariance in the population dynamics of any two species is determined by a number of factors including direct and indirect species interactions (e.g., competition effects), similarity in responses to environmental change (e.g., population responses to weather), and in the fundamental aspects governing population growth (e.g., intrinsic rate of population increase and density dependence; Birch, 1948; Loreau & de Mazancourt, 2013; Wallner, 1987; Walther et al., 2002).

If multiple species perform the same ecosystem function and decline synchronously (e.g., through strong positive correlations between response and effect traits; Suding & Goldstein, 2008), then the overall ecosystem function delivered by the species community is likely to decline, albeit just temporarily. This may lead to levels of functioning falling below some threshold that causes a socially unacceptable deficit in ecosystem services (e.g., yield deficits due to a loss of pollination function). Conversely, asynchronous dynamics of species in the same functional guild are expected to lead to more stable ecosystem functioning and subsequent ecosystem service provision (Ives, Gross, & Klug, 1999; Loreau & de Mazancourt, 2013; Yachi & Loreau, 1999).

To explore these risks to ecosystem function, in this study, we map ecosystem functions onto species “response guilds” identified through analysis of the covariance between species' historical responses to environmental change. We also explore how phylogenetic relationships between species can be related to response guilds (Díaz et al., 2013), which will lend additional understanding to species conservation and ecosystem management.

To demonstrate our method, we use butterfly time series data. Butterflies are often used as indicators for other taxonomic groups (Thomas, 2005). They perform a range of ecosystem functions that underpin supporting, regulating, and cultural services and have excellent population time series data available. Three ecosystem functions were selected to demonstrate how this new method can be used to examine the resilience of ecosystem functioning: (a) the provision of food to higher trophic levels, as lepidopteran larvae are a key food source for many bird species during chick development (Visser, Holleman, & Gienapp, 2006); (b) outcrossing pollination function, comprising the important role that butterflies play in dispersing wildflower pollen over large distances (Courtney, Hill, & Westerman, 1982); and (c) aesthetic cultural function, through members of the public experiencing culturally important taxonomic groups, which underpin cultural ecosystem services that support well‐being (Clark et al., 2014).

## 2 MATERIALS AND METHODS

### 2.1 Creating a population dynamics correlation matrix of interannual changes in abundance

UK‐wide annual abundance indices for 54 UK butterfly species from 1976 to 2014 were available from the UK Butterfly Monitoring Scheme (UKBMS). UKBMS data were collected by volunteers using the “Pollard walk” method (Pollard & Yates, 1993). Collated indices were calculated in a two‐step method. First, site abundance indices were calculated by fitting a generalized additive model to count data from each site, in order to estimate missing data values within a year (Rothery & Roy, 2001; further description can be found in Botham, Brereton, Middlebrook, Randle, & Roy, 2013). Second, the site abundance indices were used to calculate national collated indices, as with other European species monitoring schemes (ter Braak, van Strien, Meijer, & Verstrael, 1994). This was achieved using a log‐linear Poisson regression model to calculate expected counts each year, with a site factor to take into account differences between sites (UKBMS, 2016) and a year factor to account for missing years. These national‐level abundance time series reflect aggregate changes of UK populations to broad environmental conditions, such as weather effects (Roy, Rothery, Moss, Pollard, & Thomas, 2001), as well as density dependence (Pollard, Lakhani, & Rothery, 1987).

**Figure 1:**
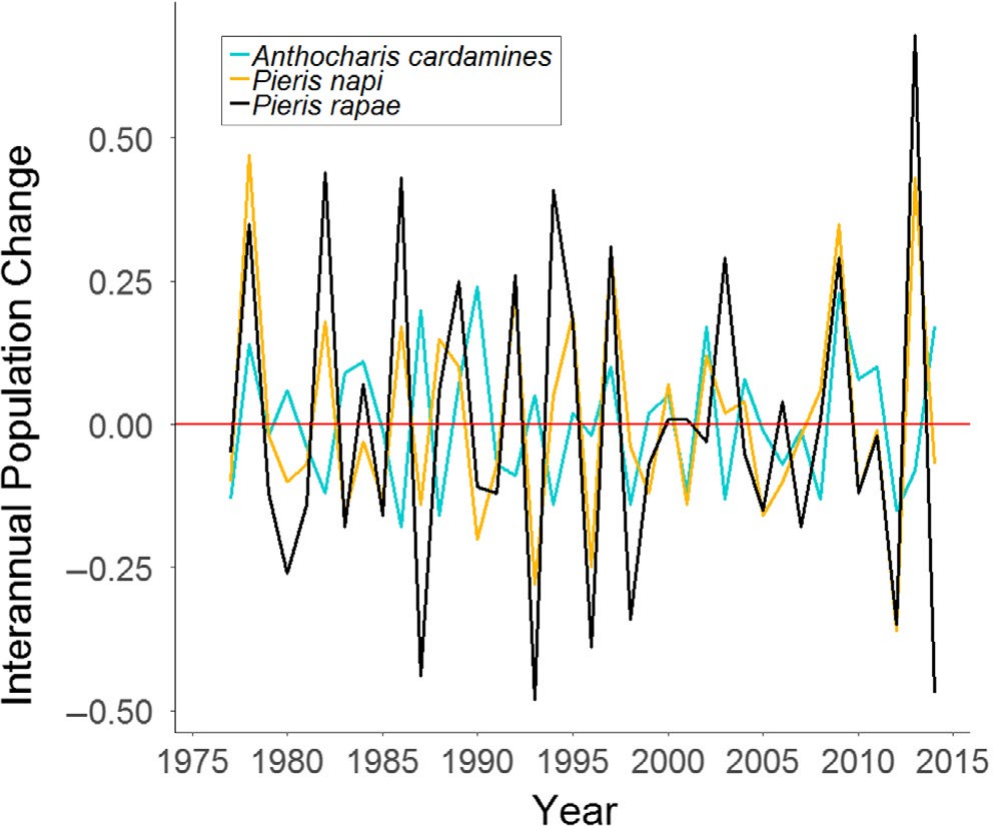
Comparison of interannual population changes for three butterfly species. Green‐veined white *Paris napi* and small white *Paris rapae* have highly correlated population dynamics (Pearson's *r* = 0.81), indicating they have responded to past environmental change in the same way. Green‐veined white *P. napi* and orange tip *Anthocharis cardamines* have much less correlated population dynamics (*r* = 0.05), indicating they respond differently to changes in the environment; that is, the same environmental drivers have different effects on the overall populations

Using these national abundance time series, for each species interannual changes were calculated by subtracting the standardized log abundance index from that of the year preceding it, creating a dataset containing the yearly changes in species abundance for all species from 1977 to 2014. Using the base R function *cor* (R Core Team, 2016), a population dynamics correlation matrix was created using Pearson's correlation coefficient, for the interannual changes in species abundance between each pair of species (Figure 1). Only complete pairs of observations were included in the correlations. The population dynamics correlation matrix was then transformed by multiplying by −1, resulting in the pairs of species with the least synchronized population dynamics having positive values (i.e., creating a distance matrix). After this transformation, all values were increased by +1. This was necessary as the methods used to perform a hierarchical cluster analysis do so using Euclidean distances between variables; therefore, negative values cannot be included. All future references to the population dynamics correlation matrix refer to this newly transformed matrix, where a value of zero indicates perfectly positively correlated interannual dynamics between species, a value of 1 indicates no correlation, and a value of 2 indicates perfect negative correlation (i.e., opposite dynamics).

**Figure 2:**
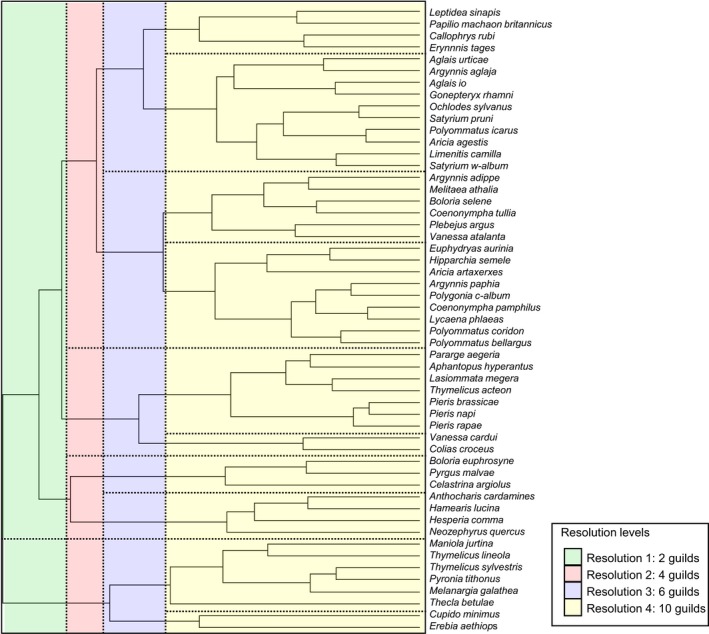
Population dynamics dendrogram showing “response guilds,” which are groups of species with similar population dynamics. Species with more correlated population dynamics join further to the right‐hand side of the dendrogram. Here, four resolutions of response guild are shown (also see Table 1), but further grouping is possible

A hierarchical cluster analysis was performed using this transformed population dynamics correlation matrix, using the *hclust* function in the program R (R Core Team, 2016). Species were grouped sequentially into clusters based upon their similarity until all species were grouped into a single cluster (R Core Team, 2016). Response guilds were then defined by plotting a dendrogram and allocating all species on a branch below a threshold into guilds (Figure 2, Table 1).

**Table 1:** Allocation of species into response guilds at different levels of resolution. Different resolutions are achieved by plotting all species onto a dendrogram and selecting species on a branch below a threshold point (see Figure 2). Species with the same number in the table are in the same response guild, meaning they tend to have more similar population dynamics (i.e., have responded to past environmental change in similar ways)

Species	Species allocation into guilds at			
	Resolution 1	Resolution 2	Resolution 3	Resolution 4
*Erebia aethiops*	1	1	1	1
*Cupido minimus*	1	1	1	1
*Thecla betulae*	1	1	1	2
*Melanargia galathea*	1	1	1	2
*Pyronia tithonus*	1	1	1	2
*Thymelicus sylvestris*	1	1	1	2
*Thymelicus lineola*	1	1	1	2
*Maniola jurtina*	1	1	1	2
*Neozephyrus quercus*	2	2	2	3
*Hesperia comma*	2	2	2	3
*Hamearis lucina*	2	2	2	3
*Anthocharis cardamines*	2	2	2	3
*Celastrina argiolus*	2	2	3	4
*Pyrgus malvae*	2	2	3	4
*Boloria euphrosyne*	2	2	3	4
*Colias croceus*	2	3	4	5
*Vanessa cardui*	2	3	4	5
*Pieris rapae*	2	3	4	6
*Pieris napi*	2	3	4	6
*Pieris brassicae*	2	3	4	6
*Thymelicus acteon*	2	3	4	6
*Lasiommata megera*	2	3	4	6
*Aphantopus hyperantus*	2	3	4	6
*Pararge aegeria*	2	3	4	6
*Polyommatus bellargus*	2	4	5	7
*Polyommatus coridon*	2	4	5	7
*Lycaena phlaeas*	2	4	5	7
*Coenonympha pamphilus*	2	4	5	7
*Polygonia c‐album*	2	4	5	7
*Argynnis paphia*	2	4	5	7
*Aricia artaxerxes*	2	4	5	7
*Hipparchia semele*	2	4	5	7
*Euphydryas aurinia*	2	4	5	7
*Vanessa atalanta*	2	4	5	8
*Plebejus argus*	2	4	5	8
*Coenonympha tullia*	2	4	5	8
*Boloria selene*	2	4	5	8
*Melitaea athalia*	2	4	5	8
*Argynnis adippe*	2	4	5	8
*Satyrium w‐album*	2	4	6	9
*Limenitis camilla*	2	4	6	9
*Aricia agestis*	2	4	6	9
*Polyommatus icarus*	2	4	6	9
*Satyrium pruni*	2	4	6	9
*Ochlodes sylvanus*	2	4	6	9
*Gonepteryx rhamni*	2	4	6	9
*Aglais io*	2	4	6	9
*Argynnis aglaja*	2	4	6	9
*Aglais urticae*	2	4	6	9
*Erynnis tages*	2	4	6	10
*Callophrys rubi*	2	4	6	10
*Papilio machaon britannicus*	2	4	6	10
*Leptidea sinapis*	2	4	6	10
*Carterocephalus palaemon*	2	4	6	10

### 2.2 Comparison of interannual population dynamics with phylogenetic relationships

**Figure 3:**
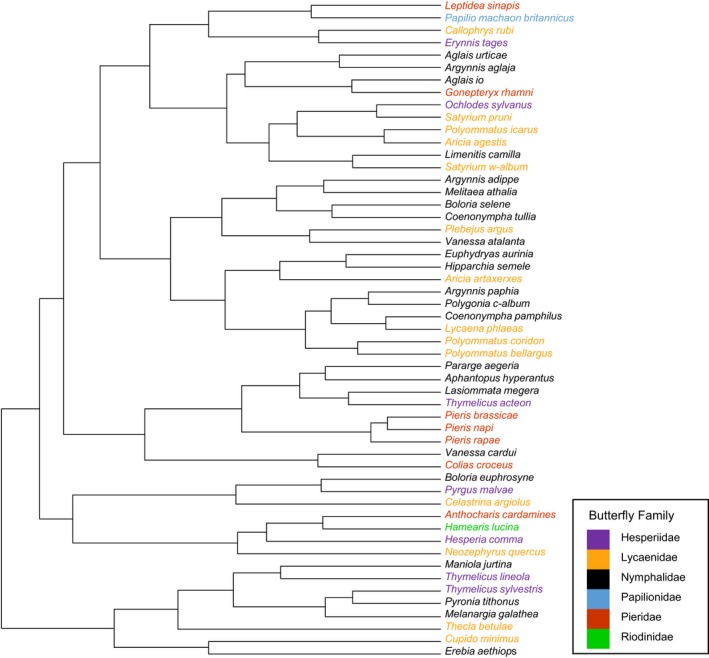
Population dynamics dendrogram with butterfly species names colored by family to show phylogenetic patterning of population dynamics. Species with more correlated population dynamics join further to the right‐hand side of the dendrogram

In order to determine whether similarities in species population dynamics are related to the genetic relatedness of species (Figure 3), a Mantel test was carried out using a matrix of genetic distances and the population dynamics correlation matrix. Using 1,000 possible phylogenies of British butterflies created by Roy et al. (2015), for each phylogeny we extracted branch lengths between all pairs of UK butterfly species using the *cophenetic* function from the *ape* package in R (Paradis, Claude, & Strimmer, 2004). Average branch lengths between each pair of species across all trees were then calculated and inputted into a matrix of phylogenetic distances. The phylogenetic and population dynamics correlation matrices were then trimmed to include only species occurring in both (*n* = 43 species in total). The similarity of the two matrices was determined via a Mantel test with 9,999 permutations, using the *mantel* function from the *ecodist* package in R (Goslee & Urban, 2007). P‐values are determined by comparing the sum of the distance values between the two matrices to the sums of randomized permutations of the matrices. Under the assumption that if the two matrices are related, the sum of their values will be high and randomization of the matrices will result in the sums being lower. *p*‐Values are calculated by dividing the number of times that the sum of the matrices is higher than the original nonrandomized matrices by the number of permutations plus the number of times the sum was higher. Further details can be found in Mantel (1967) and explained in Diniz‐Filho et al. (2013).

### 2.3 Calculating proxies of species' roles in ecosystem functioning

We combined ecological theory with published trait datasets to develop new proxies for the relative roles of UK butterfly species in delivering three broad types ecosystem functions: (a) the provision of food to higher trophic levels, (b) wildflower pollination (outcrossing) function, and (c) aesthetic cultural function. Our basic approach is to develop “production functions” that combine relevant trait data to estimate the relative roles of species in a community in contributing to ecosystem function. Beyond these broad functions, we can also calculate several “sub‐functions” (e.g., wildflower pollination function is assessed for different plant families). This approach is an extension of traditional community functional ecology approaches that often use a single trait or functional grouping as a proxy for ecosystem functioning (Funk et al., 2017; Luck et al., 2012). It allows better incorporation of basic ecological process understanding into our predictions of species' functional roles (e.g., outcrossing pollination can be a function of both insect mobility and plant association). The approach can also be extended further in light of new understanding and available data (e.g., outcrossing pollination is also likely affected by amount of pollen carried on an insect's body and the likelihood of pollen transfer during flower visitation). Thus, we see our method as a provisional approach toward more nuanced investigation of ecosystem functioning, beginning with the basic production functions below. Standardized trait values for all species can be found in Table 2.

**Table 2:** Standardized trait scores for five example traits: larval biomass, cultural function, and three levels of pollination outcrossing function. Trait scores scaled between zero and one by dividing all scores by the maximum value for that trait across all species. See main text for data sources

Species	Biomass index (B)	Cultural function index (C)	General wildflower pollination index (*P*)	Brassicaceae pollination index (*P*_Brassicaceae_)	Caryophyllaceae pollination index (*P*_Caryophyllaceae_)
*Aglais io*	0.125	0.699	0.116	0.074	0
*Aglais urticae*	0.121	0.396	0.21	0.138	0
*Anthocharis cardamines*	<0.001	0	<0.001	>0.001	0
*Aphantopus hyperantus*	0.25	0.326	0.19	0	0
*Argynnis adippe*	NA	0	NA	NA	NA
*Argynnis aglaja*	<0.001	0	<0.001	0	0
*Argynnis paphia*	0.002	0	0.002	0	0
*Aricia agestis*	<0.001	0	<0.001	0	0.001
*Aricia artaxerxes*	<0.001	0	<0.001	0	0
*Boloria euphrosyne*	<0.001	0	<0.001	0	>0.001
*Boloria selene*	<0.001	0	<0.001	0	>0.001
*Callophrys rubi*	<0.001	0	<0.001	0	>0.001
*Carterocephalus palaemon*	NA	0	NA	NA	NA
*Celastrina argiolus*	0.002	0.067	0.004	0	0
*Coenonympha pamphilus*	0.006	0	0.005	0	0.008
*Coenonympha tullia*	<0.001	0	<0.001	0	0
*Colias croceus*	<0.001	0	<0.001	0	0
*Cupido minimus*	<0.001	0	<0.001	0	0
*Erebia aethiops*	<0.001	0	<0.001	0	0
*Erynnis tages*	<0.001	0	<0.001	0	>0.001
*Euphydryas aurinia*	NA	0	NA	NA	NA
*Gonepteryx rhamni*	0.005	0.062	0.005	0.003	0
*Hamearis lucina*	NA	0	NA	NA	NA
*Hesperia comma*	<0.001	0	<0.001	0	0
*Hipparchia semele*	<0.001	0	<0.001	0	0
*Lasiommata megera*	0.001	0	0.001	0	0
*Leptidea sinapis*	<0.001	0	<0.001	0	0
*Limenitis camilla*	<0.001	0	NA	0	0
*Lycaena phlaeas*	0.003	0.059	0.005	0	0
*Maniola jurtina*	1	0.911	1	0	0
*Melanargia galathea*	0.009	0.099	0.008	0	0
*Melitaea athalia*	NA	0	NA	NA	NA
*Neozephyrus quercus*	<0.001	0	NA	0	>0.001
*Ochlodes sylvanus*	0.011	0.106	0.008	0	0.010227
*Papilio machaon britannicus*	<0.001	0	<0.001	0	>0.001
*Pararge aegeria*	0.13	0.177	0.11	0	0
*Pieris napi*	0.25	0.26	0.35	0.383	0
*Pieris brassicae*	0.612	0.923	0.627	0.250	0
*Pieris rapae*	0.561	0.985	0.898	0.561	0
*Plebejus argus*	<0.001	0	<0.001	>0.001	0
*Polygonia c‐album*	0.031	0.18	0.029	0	0
*Polyommatus bellargus*	<0.001	0	NA	0	0
*Polyommatus coridon*	<0.001	0	NA	0	0
*Polyommatus icarus*	0.017	0.173	0.027	0	0
*Pyrgus malvae*	NA	0	NA	NA	NA
*Pyronia tithonus*	0.355	1	0.325	0	0
*Satyrium pruni*	NA	0	NA	NA	NA
*Satyrium w‐album*	<0.001	0	<0.001	0	0
*Thecla betulae*	<0.001	0	<0.001	0	0
*Thymelicus acteon*	<0.001	0	<0.001	0	0
*Thymelicus lineola*	NA	0	NA	0	0
*Thymelicus sylvestris*	0.018	0	0.017	0	0
*Vanessa atalanta*	0.068	0.396	0.081	0	0
*Vanessa cardui*	0.013	0.071	NA	0	0

#### 2.3.1 Provision of food to higher trophic levels

We aimed to create an index of total butterfly larval biomass which reflects the provision of food to higher trophic levels, that is, as a food source for many bird species during chick development (Visser et al., 2006). Using updated 10 km resolution butterfly occupancy data provided by Butterfly Conservation (Asher et al., 2001; Fox et al., 2015) and abundance data from the stratified‐sampling UK Wider Countryside Butterfly Survey (WCBS), described in Brereton, Cruickshanks, Risely, Noble, and Roy (2011), we calculated an estimate for the relative average expected density of individuals across the UK. These relative national density scores were calculated using Equation 1 below, where D = relative national density of individuals, O = average number of 10km^2^ grid squares across the UK occupied by a species between 2009 and 2017, A = average number of observations for a species between 2009 and 2017 from the WCBS survey, and OA_max_ = maximum O.A score across all species. Thus, the index is standardized to scale between zero and one, with a relative national density of one for the most widely occurring species—the meadow brown *Maniola jurtina*.

(1) $$D=\left(O\text{.A}\right)/{\text{OA}}_{\max}$$

This index of relative national density was then combined with larval length data (L; in mm) described in Carter and Hargreaves (1986), to estimate the relative total butterfly biomass across the UK, under the assumptions that (a) larval length is proportionally related to larval biomass with a constant scaling factor, and (b) species with high adult abundances also have a high larval abundances and, therefore, provide more food biomass to higher trophic levels. Using Equation 2 below, a relative larval biomass score for each species was calculated, where *B* = total larval biomass index and DL_max_ = maximum D.L score across all species (*M. jurtina*).

(2) $$B=D\text{.L}/{\text{DL}}_{\max}$$

#### 2.3.2 Wildflower pollination (outcrossing) function

Pollination by butterfly species is an important source of outcrossing and maintenance of the genetic diversity of wild flowers, as many species travel further distances than other pollinators (Courtney et al., 1982). The relative national density (*D*), combined with species' mobility scores, was used as a proxy for wildflower outcrossing pollination function (*P*), under the assumption that species with a greater number of individuals, and higher levels of movement provide a greater function. Mobility indices (*M*) were taken from Cowley et al. (2001). To standardize the index between zero and one, all values were divided by the maximum D.M. score (DM_max_).

(3a) $$P=\left(D\text{.M}\right)/{\text{DM}}_{\max}$$

Additionally, we estimated pollination function for each plant family individually (*P_x_*), where *X* = 1 if a butterfly species visited the plant family or *X* = 0 if the species did not (data from Dennis, 2010; Equation 3b below). To standardize the index between zero and one, the denominator DMX_max_ reflects the maximum D.M.X score across all butterfly species for any given plant family X.

(3b) $$P_{x}=\left(D\text{.M}\text{.X}\right)/{\text{DMX}}_{\max}$$

**Figure 4:**
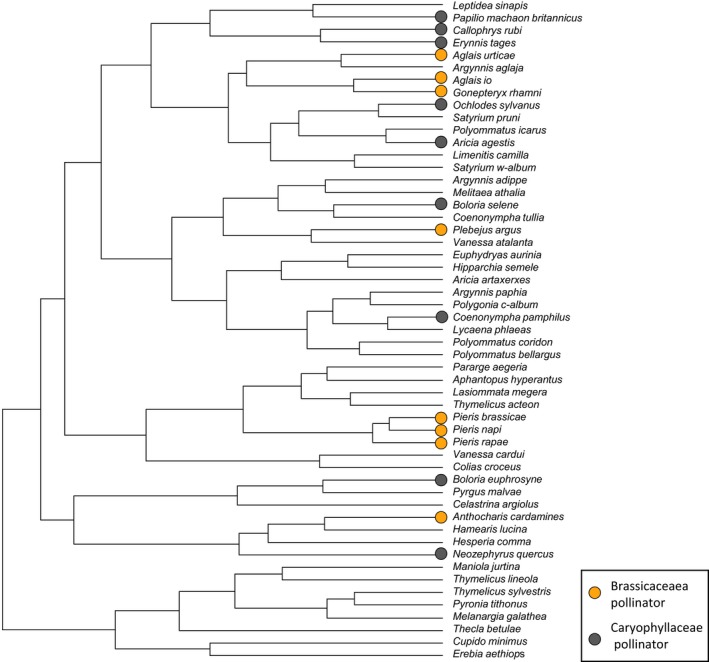
Standardized Brassicaceae and Caryophyllaceae pollination scores (*P*_x_) mapped onto the population dynamics dendrogram. Species proposed to provide a higher level of outcrossing pollination function for Brassicaceae and Caryophyllaceae are indicated by circles

For this case study, we present results for two plant families, Brassicaceae and Caryophyllaceae, chosen because each is visited by similar numbers of butterfly species (eight and nine species, respectively; Dennis, 2010), which are clustered differently across the population dynamics dendrogram (Figure 4).

#### 2.3.3 Aesthetic cultural function

Butterflies are a culturally important taxonomic group, constituting a major part of the general public's engagement with nature (Clark et al., 2014). By determining which species the general public have the highest awareness of, it is possible to estimate the level to which people may notice declines in species. For butterflies, large amounts of data are collected by skilled volunteers on UKBMS sites or WCBS squares across the wider countryside. Unlike UKBMS or WCBS transects, the Big Butterfly Count (BBC) encourages data collection by members of the general public in short 15‐min surveys over a one‐month period in summer (Dennis, Morgan, Brereton, Roy, & Fox, 2017). As a result, the survey is a better measure of the species that members of the public see most often in their local environment. Using published results from the BBC described in Dennis et al. (2017), the mean average number of recordings for the 18 most recorded UK butterfly species between 2011 and 2017 was calculated. Relative cultural function scores were calculated using Equation 4, where *C* = relative cultural function score, *Y* = individual species average score from the BBC survey, and *Y*_max_ = highest species average BBC score (gatekeeper *Pyronia tithonus*). Species that did not occur in the top 18 species in the BBC had negligible occurrence in local environments and were given a score of zero.

(4) $$C=Y/Y_{\max}$$

#### 2.3.4 Associations between ecosystem function proxies and species' response guilds

**Figure 5:**
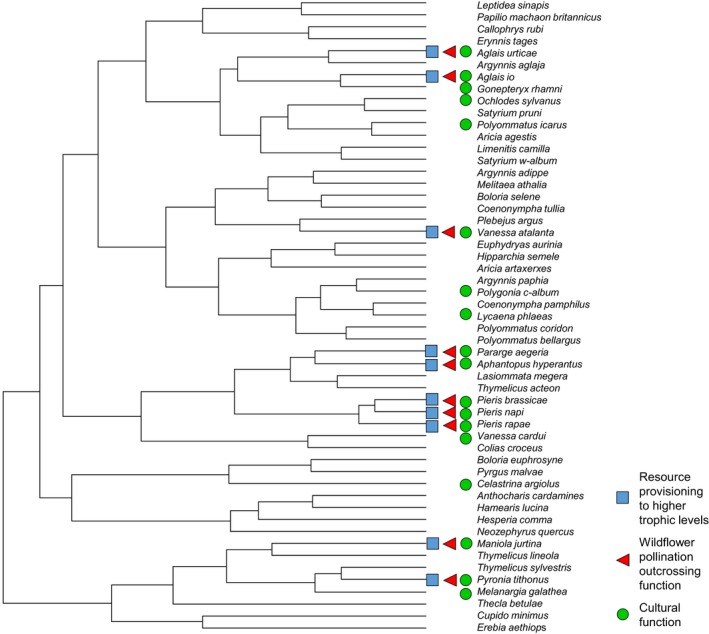
Resource provisioning to higher trophic levels, general wildflower outcrossing pollination, and cultural function scores mapped onto the population dynamics dendrogram. For resource provisioning and pollination, the ten species with the highest index scores have been mapped and are indicated by colored squares and triangles, respectively. For cultural functioning, all species with a score greater than zero have been mapped and are indicated by green circles

Species' scores for their relative role in providing different ecosystem functions were mapped onto the population dynamics dendrogram, showing which species provided the highest levels of functioning and where they clustered (Figures 4 and 5). In order to determine whether functionally important species were distributed nonrandomly across the population dynamics dendrogram, the differences in scaled (unit variance and zero mean) ecosystem function scores between all pairs of UK butterfly species were calculated and absolute values were inputted into a matrix of Euclidean distance. Each ecosystem function score matrix then underwent a Mantel test, as described previously, with the transformed population dynamics correlation matrix to determine whether the two showed significant associations.

## 3 RESULTS

### 3.1 Comparison of interannual population dynamics with phylogenetic relatedness

The results of the Mantel test show that increasing values in the transformed population dynamics correlation matrix are significantly positively associated with increasing genetic distances between species (*p* < .05, Table 3). Therefore, the greater the genetic distance between two species, the greater the difference in their population dynamics, suggesting that closely related species respond more similarly to environmental change than more distantly related species (*r* = 0.151; Table. 3); that is, in UK butterflies, we find there to be significant heritability in species' population dynamics.

**Table 3:** Mantel test results relating differences in butterfly population dynamics, genetic distances matrix, and all trait matrices

Matrix 1	Matrix 2	Observed correlation (Mantel r)	Significance (simulated *p*‐value)	Lower confidence limit (2.5%)	Upper confidence limit (97.5%)
Population dynamics	Phylogenetic tree	0.143	.003	0.100	0.185
Population dynamics	Larval biomass	−0.279	.868	−0.567	0.089
Population dynamics	Cultural function	0.086	.141	−0.006	0.157
Population dynamics	General wildflower pollination score	−0.162	.665	−0.517	0.198
Population dynamics	Brassicaceae pollination score	−0.232	.663	−0.419	0.000
Population dynamics	Caryophyllaceae pollination score	0.489	.163	0.000	0.780

### 3.2 Comparing trait distributions with population dynamics

There were no significant associations between the transformed population dynamics correlation matrix and either the larval biomass or cultural function matrices (*p* = .868 and *p* = .141, respectively [Table 3]). Additionally, none of the matrices of pollination functioning (general wildflower pollination, Brassicaceae or Caryophyllaceae) showed any significant associations with the population dynamics correlations (*p* = .665, *p* = .663, and *p* = .163, respectively [Table 3]). Therefore, functionally important species are not patterned across the dendrogram in a manner significantly different from random for any of the traits investigated; that is, they are not significantly clustered within response guilds.

## 4 DISCUSSION

The need to predict the effects of environmental change on ecosystem services remains an urgent priority (De Palma et al., 2017; Díaz et al., 2013; Oliver et al., 2015). Previous methods have so far failed to adequately address this priority, and a fresh perspective is required to overcome the decades‐long impasse (Díaz & Cabido, 1997; Funk et al., 2017; Lavorel & Garnier, 2002). In this paper, we have demonstrated an alternative method that begins to overcome some of the previous constraints, by using long‐term monitoring data to inform on overall species' responses to past environmental change (i.e., integrated across multiple aspects of historic environmental change). This eliminates the need to ascertain relationships between individual response and effects traits, and combine these additively in order to understand overall responses to multivariate environmental change and the subsequent effects on function. Using long‐term monitoring data, we show that correlations between species' population dynamics can be used to determine whether functionally important species respond to historic environmental drivers in the same way, which according to theory should inform on the resilience of ecosystem functioning (Lavorel & Garnier, 2002; Loreau & de Mazancourt, 2013; Oliver et al., 2015). Essentially, rather than considering the correlations between individual response and effect traits, we consider the correlation between ecosystem function proxies and “response guilds,” in order to predict ecosystem service resilience.

Applying this approach for three types of ecosystem function that underpin supporting, regulating, and cultural services provided by UK butterflies, we found that provision of food for higher trophic levels, wildflower pollination function, and aesthetic cultural function appear relatively resilient to environmental change. These functional traits were spread across a number of response guilds, suggesting uncorrelated or even asynchronous responses of functionally important species, which should lead to more stable ecosystem functioning (Loreau & de Mazancourt, 2013; Mori, Furukawa, & Sasaki, 2013) and lower levels of ecosystem function deficit (Allan et al., 2011; Oliver et al., 2015). The investigation into the stability of wildflower pollination function showed that butterfly species that visit the family Caryophyllaceae showed more clustering into response guilds than those that are important for Brassicaceae pollination, perhaps suggesting a greater resilience of pollination of the latter, although in both cases the overall correlation between ecosystem function and population dynamics matrices was not significant.

We propose that a higher number of functionally important species across multiple response guilds lead to more resilient ecosystem functioning. Therefore, any species which is the sole representative of a response guild should be more important for resilience, as these species have asynchronous dynamics compared with others and so will have more influence on the statistical averaging (“portfolio”) effect that results in an overall more stable ecosystem function from a community (Ives et al., 1999; Tilman, 1999; Yachi & Loreau, 1999). Using cultural function in UK butterflies as an example, we find that in some cases, multiple functionally important species are aggregated into the same response guild, for example, *Pieris rapae, Pieris napi, Pieris brassicae, Aphantopus hyperantus,* and *Pararge aegeria* (Figure 5, Table 1). In other cases, however, important functional species are isolated in their own response guilds, for example, the holly blue butterfly *Celastrina argiolus* (Figure 5, Table 1). We suggest that this species is particularly important because in years when the other species are in synchronized decline, this may be one of the few remaining species apparent in gardens, ensuring at least some butterflies are seen and providing the maintenance of cultural services. Populations of this species appear to respond to an interacting set of drivers related to weather and parasitoids in a unique way (Oliver & Roy, 2015).

In our analysis of UK butterflies, we found that population dynamics show some degree of heritability, with species more closely related more likely to respond to environmental drivers in the same way (Figure 3). This fits with the niche conservatism theory proposed by Harvey and Pagel (1991), whereby closely related species are more likely to be ecologically similar (Ackerly, 2009). Interestingly, it contrasts with results from Diamond, Frame, Martin, and Buckley (2011) who found little evidence of a phylogenetic signal in UK butterflies' phenological responses. Our findings of a phylogenetic patterning in population dynamics suggest there might be a potential opportunity for conservationists to infer how rarer, data‐sparse species respond to environmental change based on the responses of related species for which population dynamics data are available.

Although we believe our methodology offers significant advances over previous reductionist approaches for predicting resilience of ecosystem functioning in real‐world situations, it has several limitations. First, our method is most applicable to species for which long‐term monitoring data are available; for example, in the UK, this primarily comprises groups such as plants, butterflies, birds, aphids, moths, and ground beetles, for example, Morecroft et al. (2009). Other spatially replicated standardized recording schemes, such as for pollinators, are still in their infancy, although should produce usable data for this method in due course (Hayhow et al., 2016; Pocock, Roy, Preston, & Roy, 2015). Furthermore, as well as an expansion in population monitoring schemes, there has also been a recent increase in the taxonomic coverage and participation in citizen science distribution recording schemes (Pocock, Tweddle, Savage, Robinson, & Roy, 2017). In some cases, yearly changes in the total number of biological records (georeferenced records of a species presence at a particular time) can be used as a proxy for yearly changes in species' abundance, as shown by Mason et al., (2018). Using such proxies for time series data would open up this method to a far greater range of species and ecosystem functions, greatly increasing its potential implementation.

Second, using our approach to predict resilience of ecosystem functioning in the future requires the assumption that patterns of species' covariance will remain similar over time. This is a reasonable assumption to some degree since morphological and physiological traits determine responses to environmental change (supported by our result reflecting significant heritability), and such traits can only change relatively slowly through evolution. However, it remains feasible that newly arising environmental drivers of change could affect individual species idiosyncratically, for example, a newly arriving pathogen which is species‐specific. Therefore, some deliberation is needed with regard to the appropriate level of uncertainty when making predictions, as in any ecological forecasting attempt (Oliver & Roy, 2015).

Finally, there are still constraints in applying these methods based on the availability of functional “effect” traits. To demonstrate the applicability of the method, we used three basic proxies for ecosystem functions delivered by butterflies. Uncertainty remains in the appropriateness of these proxies; for example, we assume that all species found in urban gardens have equal cultural value, with total cultural function scaling proportionally with relative butterfly density. However, certain species might be more culturally important than others (Hiron, Pärt, Siriwardena, & Whittingham, 2018), and there may be diminishing marginal returns of cultural value with increasing butterfly abundance. While such concerns are not critical in demonstrating the applicability of the method, further refinement of trait selection and calculation will be necessary for using this method for conservation strategies and in predictive frameworks. Nevertheless, our approach needs far less trait specific information than previous reductionist approaches, because we bypass the need to assess response traits for every species and for multiple different aspects of environmental change. Finally, in this study, we have not proposed levels of asynchrony in population dynamics below which “safe” thresholds of ecosystem function resilience are passed, and further work is necessary, incorporating social science research into levels of acceptable environmental risk.

In summary, while there remains uncertainty in the links between species traits, population changes, and ecosystem function, our method is more practical and feasible than previous reductionist approaches. It uses long‐term monitoring data based on co‐varying species' responses to multiple aspects of environmental change, and we hope it offers a significant advancement in our ability to predict ecosystem function resilience.

## AUTHOR CONTRIBUTIONS

THO conceived the study with input from MPG; DBR and TB collated and processed the data. MPG performed the analysis. All authors contributed to the writing of the manuscript.

## Data Availability

We will not be archiving data because all data used in this manuscript have already been published or archived elsewhere.
